# Higher tissue concentrations of cefuroxime achieved with preoperative stand-alone intraosseous regional prophylaxis in robotic-assisted total knee arthroplasty: a randomized controlled trial

**DOI:** 10.1186/s42836-026-00436-0

**Published:** 2026-10-19

**Authors:** Te Liu, Heng Zhang, Ming Guo, Runkai Zhao, Ye Tao, Zhuo Zhang, Guoqiang Zhang, Jun Fu, Ming Ni

**Affiliations:** 1 Senior Department of Orthopedics, the Fourth Medical Center of Chinese PLA General Hospital, Beijing, 100036 China; 2 Department of Orthopedics, Peking Union Medical College Hospital, Chinese Academy of Medical Sciences & Peking Union Medical College, Beijing, 100730 China; 3 Medical School of Chinese PLA, Beijing, 100853 China; 4 The First Affiliated Hospital of Henan Medical University, Xinxiang, 453100 China

**Keywords:** Robotic-assisted total knee arthroplasty (RA-TKA), Intraosseous regional administration (IORA), Cefuroxime, Prophylactic antibiotics, Tissue concentration, Systemic concentration, Periprosthetic joint infection (PJI)

## Abstract

**Background:**

Intraosseous regional administration (IORA) of antibiotics can achieve higher local tissue concentrations than intravenous administration in conventional total knee arthroplasty (TKA). This study aimed to evaluate the tissue concentration and systemic concentration of preoperative stand-alone intraosseous cefuroxime in robotic-assisted total knee arthroplasty (RA-TKA) and identify a candidate dose for further evaluation.

**Methods:**

Thirty-nine patients undergoing MAKO RA-TKA were randomly allocated in a 1:1:1 ratio to receive intraosseous cefuroxime 750 mg (R750_IO_), intraosseous cefuroxime 1,500 mg (R1500_IO_), or intravenous cefuroxime 1,500 mg (R1500_IV_). No additional intravenous antibiotics were administered before incision or during surgery. During surgery, nine tissue samples were collected: bone samples from the femur or tibia, synovial and fat samples from the surgical area, and systemic blood samples twice for cefuroxime level detection. Intraoperative vital signs, inflammatory markers on days 1 and 3 postoperative, and complications within 3 months were recorded.

**Results:**

The final analysis included 38 patients: 13 in the R750_IO_ group, 13 in the R1500_IO_ group, and 12 in the R1500_IV_ group. Compared with the R1500_IV_ group, both R1500_IO_ and R750_IO_ groups showed significantly higher tissue concentrations (*p* < 0.05) but lower initial blood concentrations (*p* < 0.05). While the R1500_IO_ group had higher tissue concentrations than the R750_IO_ group, its post-tourniquet-release blood concentration was significantly higher (*p* = 0.005). No significant differences were found in postoperative inflammatory markers and intraoperative vital signs among the three groups (*p* > 0.05), and no complications occurred within 3 months.

**Conclusions:**

Despite multiple intramedullary pins and longer operative time in robot-assisted TKA, a single intraosseous dose of cefuroxime still achieved high tissue concentrations. Compared to conventional intravenous infusion of 1,500 mg cefuroxime, preoperative stand-alone intraosseous administration of either 750 mg or 1,500 mg cefuroxime achieved higher local tissue concentrations. Moreover, the 750 mg intraosseous regimen was associated with a lower post-tourniquet-release systemic concentration than the 1,500 mg intraosseous regimen, supporting its further clinical evaluation as a candidate dose in RA-TKA.

## Introduction

Despite efforts to prevent infections, periprosthetic joint infection (PJI) after primary total knee arthroplasty (TKA) remains a significant challenge [1–4]. Bacterial contamination during TKA surgery is unavoidable due to inherent environmental and skin bacteria, even with strict aseptic techniques [5]. The rational use of prophylactic antibiotics is the most responsible measure for preventing infections [5–7], with efficacy dependent on tissue antibiotic concentrations exceeding the minimum inhibitory concentration (MIC) of the target pathogens and can be maintained from the time of skin incision to wound closure [8]. Coagulase-negative staphylococci (CONS) and *Staphylococcus aureus* cause up to two-thirds of global PJIs approximately [8, 9]. Although using cephalosporins demonstrates efficacy in TKA infection prophylaxis, emerging antimicrobial resistance presents challenges: CONS resistance to cephalosporins has risen significantly, with data showing a potential near-100-fold increase in cephalosporin MIC values [10]. Preoperative intravenous antibiotic administration fails to provide this level of local antibiotic concentration [11], and such subtherapeutic levels will further drive resistance development [11–13].

Intraosseous regional administration (IORA) of vancomycin for TKA infection prevention has been validated [1, 4, 5, 14–16], providing higher local tissue concentrations while reducing systemic exposure. However, current evidence indicates that intraosseous vancomycin administration may be prioritized for high-risk PJI patients to maximize clinical benefits, whereas cephalosporins remain the primary prophylactic antibiotics for TKA infections [17, 18]. According to the 2018 International Consensus on Musculoskeletal Infection, cefazolin or cefuroxime (first- and second-generation cephalosporins, respectively) are the most commonly used and recommended perioperative prophylactic antibiotics for primary TKA [19]. Notably, in most prior studies, an additional preoperative intravenous antibiotic dose is administered alongside the IORA antibiotic for prophylactic protection. However, this dual-antibiotic approach in intraosseous (IO) procedures increases overall antibiotic consumption and negates the potential time and labor savings typically associated with IO antibiotic administration [4, 20, 21]. In our previous randomized trial involving patients undergoing conventional manual TKA, preoperative stand-alone intraosseous cefuroxime produced higher periarticular tissue concentrations than intravenous administration, while the 750 mg intraosseous regimen was associated with lower systemic concentrations than the 1,500 mg intraosseous regimen [22].

In recent years, robotic-assisted total knee arthroplasty (RA-TKA) has been increasingly adopted due to its enhanced bone resection precision, better implant matching, and gap balancing [23–26]. However, the risk of infection in RA-TKA may increase with the movement of people in the operating room, additional equipment, and longer surgery times [3, 27, 28]. Data have shown that the incidence of PJI in RA-TKA is higher than that in conventional arthroplasty [28, 29]. Additionally, the robotic technique carries a specific risk of pin tract infection [30, 31]. Therefore, enhanced infection prevention measures are particularly crucial in RA-TKA. Compared with conventional manual TKA, RA-TKA introduces several workflow-related factors that may influence the regional distribution and persistence of intraosseously administered cefuroxime. The tibial tracker pins are inserted near the proximal tibial intraosseous access region after IORA and are subsequently removed after implant placement, potentially creating additional pathways for fluid egress later in the procedure. RA-TKA also avoids the femoral intramedullary alignment rod used in conventional TKA and therefore preserves the central femoral canal. Differences in medullary canal integrity, medullary bleeding, and local fluid dilution may consequently affect regional drug distribution [32–35]. Finally, previous pharmacokinetic studies have shown that tourniquet inflation can impair periarticular antibiotic distribution and that tissue antibiotic concentrations decline over time after administration [13]. Given the generally longer operative and tourniquet times associated with RA-TKA, these workflow-specific differences raised uncertainty regarding whether the high periarticular concentrations achieved with intraosseous cefuroxime could be sustained throughout the robotic procedure [36]. Our previous study characterized the concentration profile of stand-alone preoperative intraosseous cefuroxime in conventional TKA [22]. However, it remained unclear whether these findings could be reproduced in the distinct workflow of RA-TKA. To our knowledge, intraosseous antibiotic prophylaxis has not previously been evaluated specifically in this setting. The increasing adoption of RA-TKA further underscores the clinical relevance of addressing these uncertainties [25, 30].

This study aimed to determine whether intraosseous cefuroxime in RA-TKA reliably achieves tissue concentrations, to compare the tissue and systemic concentration profiles of stand-alone preoperative IORA versus intravenous administration, and to explore dose-related concentration differences. We hypothesize that intraosseous cefuroxime in RA-TKA can provide high tissue cefuroxime concentrations and that intraosseous administration of 750 mg cefuroxime would achieve higher local tissue concentrations than intravenous infusion of 1,500 mg cefuroxime in RA-TKA.

## Material and methods

### Study design

This was a prospective, randomized, single-blind, controlled study involving patients who underwent MAKO robotic-assisted TKA surgery and received intraosseous or intravenous cefuroxime administration. This RA-TKA cohort was conducted within the same broader registered research programme as our previously reported conventional TKA cohort [22]. The programme was registered with the Chinese Clinical Trial Registry before participant recruitment (ChiCTR2400080681; registered on 4 February 2024). The two cohorts used the same dosing groups, sampling framework, analytical platform, and exploratory concentration benchmark but were recruited separately during different periods, with no overlap in participants, tissue or blood specimens, laboratory measurements, or outcome data. This study was approved by the Ethics Committee of our hospital, and written informed consent was obtained from all participants enrolled.

### Participant recruitment

This study planned to include 39 individuals diagnosed with knee osteoarthritis who underwent MAKO robotic-assisted TKA surgery between July 2024 and August 2024. The inclusion criteria encompassed: (1) patients over the age of 18 years undergoing primary TKA due to knee osteoarthritis and (2) subjects who fully understand the benefits and risks of this trial, are willing to participate in and complete the related experimental content, and have signed the informed consent form. The exclusion criteria were (1) history of compartment syndrome; (2) use of cephalosporins within seven days; (3) known allergy to cephalosporins; (4) presence of severe heart, liver, or kidney diseases; and (5) patients who had an existing infection at the surgical site or systemic infection.

### Randomization and blinding

Patients were randomly assigned to intraosseous administration of 750 mg cefuroxime (R750_IO_ group), intraosseous administration of 1,500 mg cefuroxime (R1500_IO_ group), and intravenous administration of 1,500 mg cefuroxime (R1500_IV_ group) in a 1:1:1 ratio using random numbers generated by SPSS Statistics Version 27.0 (IBM, Armonk, New York, USA), with the allocation concealed in envelopes. Participants and all laboratory personnel were blinded to group allocation.

### Antimicrobial prophylaxis protocol

**Fig. 1:**
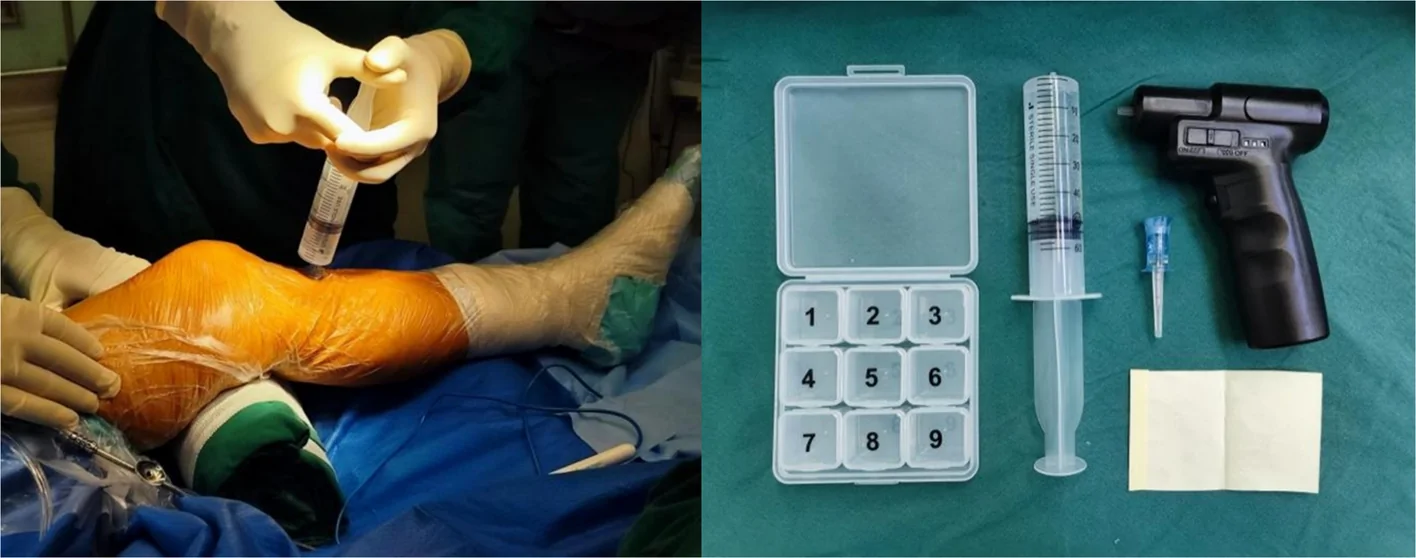
Intraosseous administration of cefuroxime (750 mg or 1,500 mg) during robotic-assisted TKA is illustrated. The injection procedure is depicted on the left, with the access point located at the proximal tibial metaphysis, approximately two fingerbreadths distal to the superior border of the tibial tubercle and one fingerbreadth medial to it. The required equipment—including an electric bone drill, bone needle, syringe, sterile dressing, and specimen box—is shown on the right

The R750_IO_ group: Nurses prepared 750 mg of cefuroxime dissolved in 50 mL of physiological saline. The IORA was performed before skin incision, following routine draping and inflation of the tourniquet in RA-TKA. A single proximal tibial intraosseous access site was used for antibiotic administration (Fig. 1). The access point was located at the proximal tibial metaphysis, approximately two fingerbreadths distal to the superior border of the tibial tubercle and one fingerbreadth medial to it. The electric bone drill was connected to the intraosseous needle and placed perpendicular to the cortical surface. A syringe containing 50 mL of cefuroxime solution was then connected, and the entire antibiotic dose was administered through this single tibial site via IO (Fig. 1). After injection, the intraosseous needle was removed, and the puncture site was covered with a sterile dressing to prevent leakage of the liquid.

The R1500_IO_ group: Intraosseous administration of 1,500 mg cefuroxime using the method described above.

The R1500_IV_ group: Intravenous infusion of 1,500 mg cefuroxime via the antecubital vein, administered preoperatively according to the hospital’s actual clinical infusion time.

In this study, “stand-alone” refers specifically to the randomized preoperative prophylactic regimen. Patients in the R750_IO_ and R1500_IO_ groups received 750 mg and 1,500 mg of cefuroxime intraosseously, respectively, whereas those in the R1500_IV_ group received 1,500 mg of cefuroxime intravenously; no additional systemic antibiotics were administered before incision or intraoperatively. No visible leakage from either the intraosseous access site or the tracker-pin sites was observed intraoperatively, including after tracker-pin insertion and removal. After surgery, all patients commenced routine postoperative intravenous antibiotic continuation within 24 h according to institutional protocol and also received standard anticoagulation treatment.

### Surgical techniques

All patients underwent standard unilateral TKA via a medial parapatellar approach with cemented posterior stabilized prostheses. A tourniquet placed at the root of the thigh was inflated to 260 to 300 mmHg at the start of the surgery, rapidly and immediately deflated after cementing. Patients underwent robotic-assisted TKA based on preoperative planning with CT using the MAKO robotic-assisted system (MAKO Robotic Arm Interactive Orthopaedic System) (Stryker, Mahwah, NJ, USA). Preoperative CT scans were used to create a three-dimensional (3D) model of the knee for surgical planning. After completion of IORA, two tibial fixation pins were inserted using an electric drill at a position four fingerbreadths distal to the tibial tubercle and 10 mm medial to it, after which a tibial tracker was mounted on the pins. Following exposure, two femoral fixation pins were placed at a location four fingerbreadths proximal to the patella and 10 mm medial to it, and a femoral tracker was mounted on the fixation pins. The fixation pins had a diameter of 3.2–4.0 mm and were all placed in a medial-to-lateral direction at a 45° angle for bicortical fixation. After registration and system evaluation of the soft tissue tension and flexion–extension gaps of the knee, distal femoral and proximal tibial osteotomies were performed. The femoral and tibial trackers and pins were subsequently removed after the prosthesis was placed.

### Specimen collection and testing

A total of nine tissue samples (comprising adipose tissue, synovium, and cancellous bone) were collected during the surgical procedure. The specific timing and anatomical sites of collection are detailed in Table 1. All tissue specimens, measuring approximately 0.5 to 1.0 cm^3^, were rinsed with physiological saline to remove excess blood and stored individually in separate containers (Fig. 1). Blood samples (2–3 mL) were collected after skin incision and following tourniquet deflation.

**Table 1:** Sampling protocol

Timing of specimens	Specimen collection site	Sample
After skin incision	Surgery Area	Subcutaneous fat 1
	Surgery Area	Synovium 1
	Forearm vein^a^	Blood sample A
Bone cut	Distal femoral	Bone 1
	Proximal tibia	Bone 2
Before implant cementing	Surgery Area	Subcutaneous fat 2
	Surgery Area	Synovium 2
	Intercondylar femoral^b^	Bone 3
After tourniquet deflation	Surgery Area	Subcutaneous fat 3
	Surgery Area	Synovium 3
	Forearm vein^a^	Blood sample B

^a^Forearm vein on the non-intravenous infusion side

^b^Intercondylar femoral opening in the distal femur

All samples were labeled anonymously using pseudo-patient identifiers and stored at − 80 °C until analysis. Samples were prepared in duplicate and analyzed for cefuroxime concentrations using liquid chromatography-tandem mass spectrometry (LC–MS/MS) according to the methods described by Young et al. and a method previously described in our earlier work [11, 14, 22].

### Follow-up and data collection

Patients were generally discharged within three to five days after surgery. Follow-up assessments were conducted at 1 day, 3 days, 30 days, and 3 months postoperatively. Vital signs were recorded 5–10 min after intraosseous regional administration (IORA) and following tourniquet deflation. Laboratory indicators, including serum C-reactive protein (CRP), interleukin-6 (IL-6), and erythrocyte sedimentation rate (ESR), were measured on the 1 and 3 postoperative days. Complications and adverse events occurring within three months after surgery were also documented.

### Data analyses and sample sizes

Statistical analyses were performed using SPSS Statistics Version 27.0 (IBM Corporation, Armonk, New York, USA). Repeated-measures analysis of variance (ANOVA) was employed to compare cefuroxime concentrations across tissue and blood samples, with age, body mass index, and surgery duration included as covariates to assess their potential influence. The Type III test of fixed effects indicated that none of the covariates had a significant impact. For pairwise comparisons of cefuroxime concentrations between treatment groups at each prespecified sampling point, independent-samples t-tests were used for normally distributed data, whereas Mann–Whitney U tests were used for non-normally distributed data. For quantitative baseline demographic variables, intraoperative vital-sign changes, and postoperative laboratory outcomes, overall comparisons among the three groups were performed using one-way analysis of variance or the Kruskal–Wallis test, as appropriate. If a significant overall difference was identified, post hoc pairwise comparisons with Bonferroni adjustment were planned. Fisher’s exact test was used for the analysis of count data. A *p*-value < 0.05 was considered statistically significant.

The sample-size calculation was based solely on the prespecified primary outcome of local tissue cefuroxime concentration. Based on prior IORA data for power calculation [11], the mean cefuroxime concentrations in fat were 186 μg/g (SD ± 109) for the intraosseous group and 11 μg/g (SD ± 5) for the intravenous group, while in bone tissue, the concentrations were 130 μg/g (SD ± 132) and 11 μg/g (SD ± 5), respectively. With a superiority margin set at ≥ 4 μg/g, a significance level of 5% (α = 0.05), and a statistical power of 90% (β = 0.10), the sample size calculated based on the difference in bone tissue concentrations was sufficient to compare the differences in cefuroxime concentrations in soft tissue. Considering a 10% loss to follow-up rate, the sample size was determined to be 13 patients per group. This aligns with power estimations reported in previous literature [11, 14, 22, 37, 38]. This calculation was not intended to provide adequate power for secondary clinical outcomes, including PJI, wound complications, inflammatory markers, or adverse events.

## Results

**Fig. 2:**
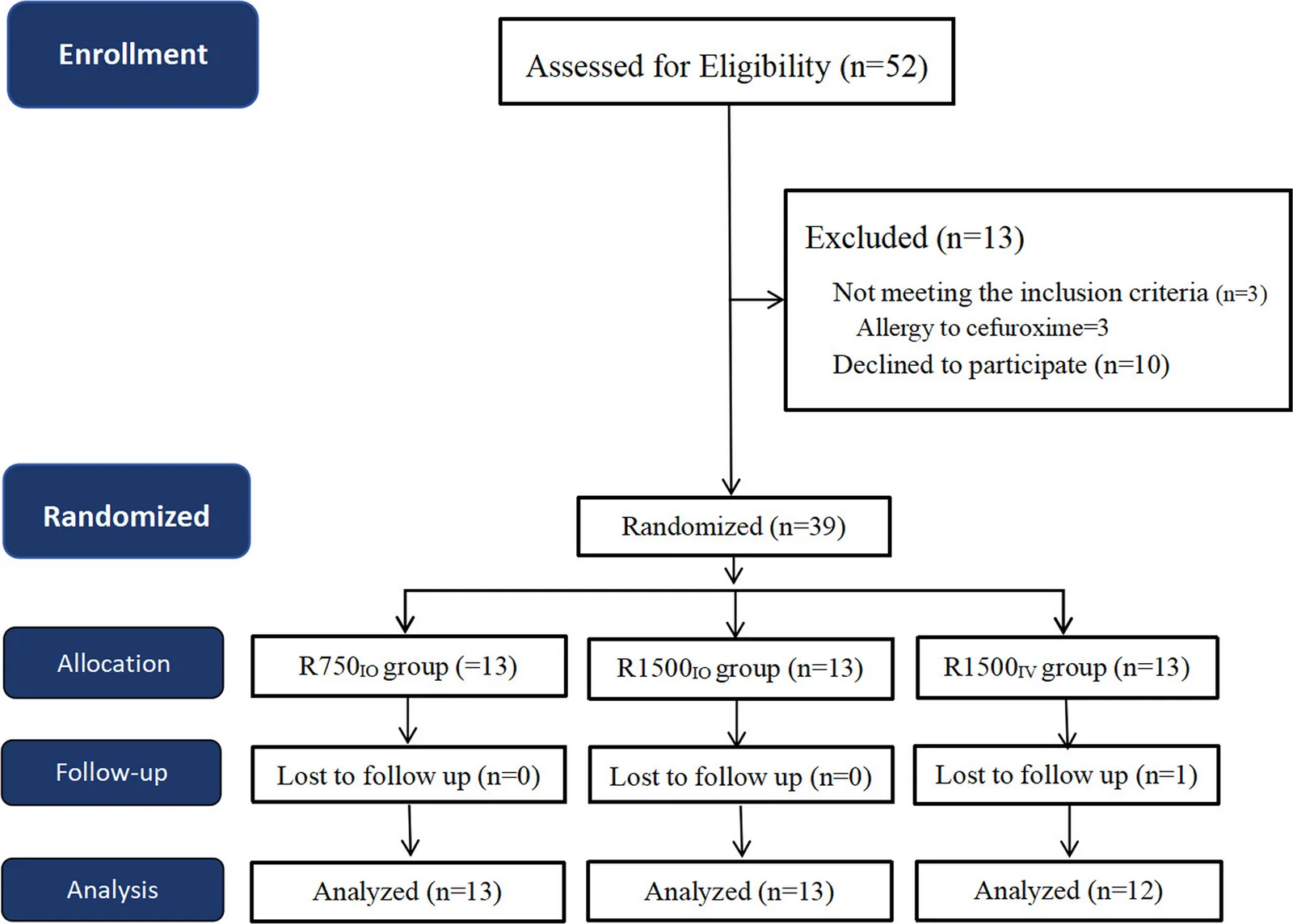
Patient randomization flowchart

From July 2024 to August 2024, a total of 39 patients were randomized equally to the R750_IO_ group (*n* = 13), R1500_IO_ group (*n* = 13), or R1500_IV_ group (*n* = 13). The prespecified enrollment target of 13 patients per group was achieved. However, one participant in the R1500_IV_ group was lost to follow-up for personal reasons after randomization and was not included in the final analysis. Therefore, 38 patients were analyzed: 13 in the R750_IO_ group, 13 in the R1500_IO_ group, and 12 in the R1500_IV_ group (Fig. 2). The preoperative antibiotic infusion duration was longer in the R1500_IV_ group compared to the other two intraosseous groups. No other statistically significant differences in the patients’ baseline demographics were observed among the three groups (*p* > 0.05). Patient baseline data are shown in Table 2.

**Table 2:** Patient demographics and clinical findings

Characteristics	R750_IO_ (*n* = 13)	R1500_IO_ (*n* = 13)	R1500_IV_ (*n* = 12)	*p*-value
Mean age, yrs (range)	64(49 to 73)	63(55 to 72)	67(60 to 74)	0.20
Mean BMI, kg/m^2^ (SD)	28.4 ± 3.6	27.1 ± 3.0	28.7 ± 3.5	0.43
**Sex, n (%)**				0.89
Male	3 (23.1)	4 (30.8)	2 (16.7)	
Female	10 (76.9)	9 (69.2)	10 (83.3)	
**ASA grade (Ⅰ to Ⅲ), n (%)**				0.32
I	0 (0)	1 (7.7)	0 (0)	
II	11 (84.6)	12 (92.3)	12 (100)	
III	2 (15.4)	0 (0)	0 (0)	
Smoking, n (%)	1 (7.7)	3 (23.1)	1 (8.3)	0.59
Diabetes, n (%)	4 (30.8)	3 (23.1)	2 (16.7)	0.89
History of knee surgery, n (%)	5 (38.5)	4 (30.8)	3 (25.0)	0.91
Tourniquet time, min (SD)	80.7 ± 9.2	83.5 ± 12.8	77.4 ± 15.9	0.59
Surgical time, min (SD)	113.4 ± 9.9	114.1 ± 19.0	112.8 ± 18.8	0.99
Antibiotic infusion time^a^, min (SD)	1.4 ± 0.5	1.8 ± 0.6	15.1 ± 7.4	< 0.001^b^

*BMI* body mass index, *ASA* American Society of Anesthesiologists, *SD* standard deviation

^a^The duration of preoperative antibiotic administration in the R1500 _IV_ group refers to the time from initiation to completion of cefuroxime infusion, whereas in the other two groups, it refers to the duration of intraosseous administration. The shorter duration in IO groups reflects the rapid bolus administration through the intraosseous route, which is consistent with the standard IORA technique

^b^Indicates statistical significance

### Cefuroxime concentrations in tissue and blood samples

**Fig. 3:**
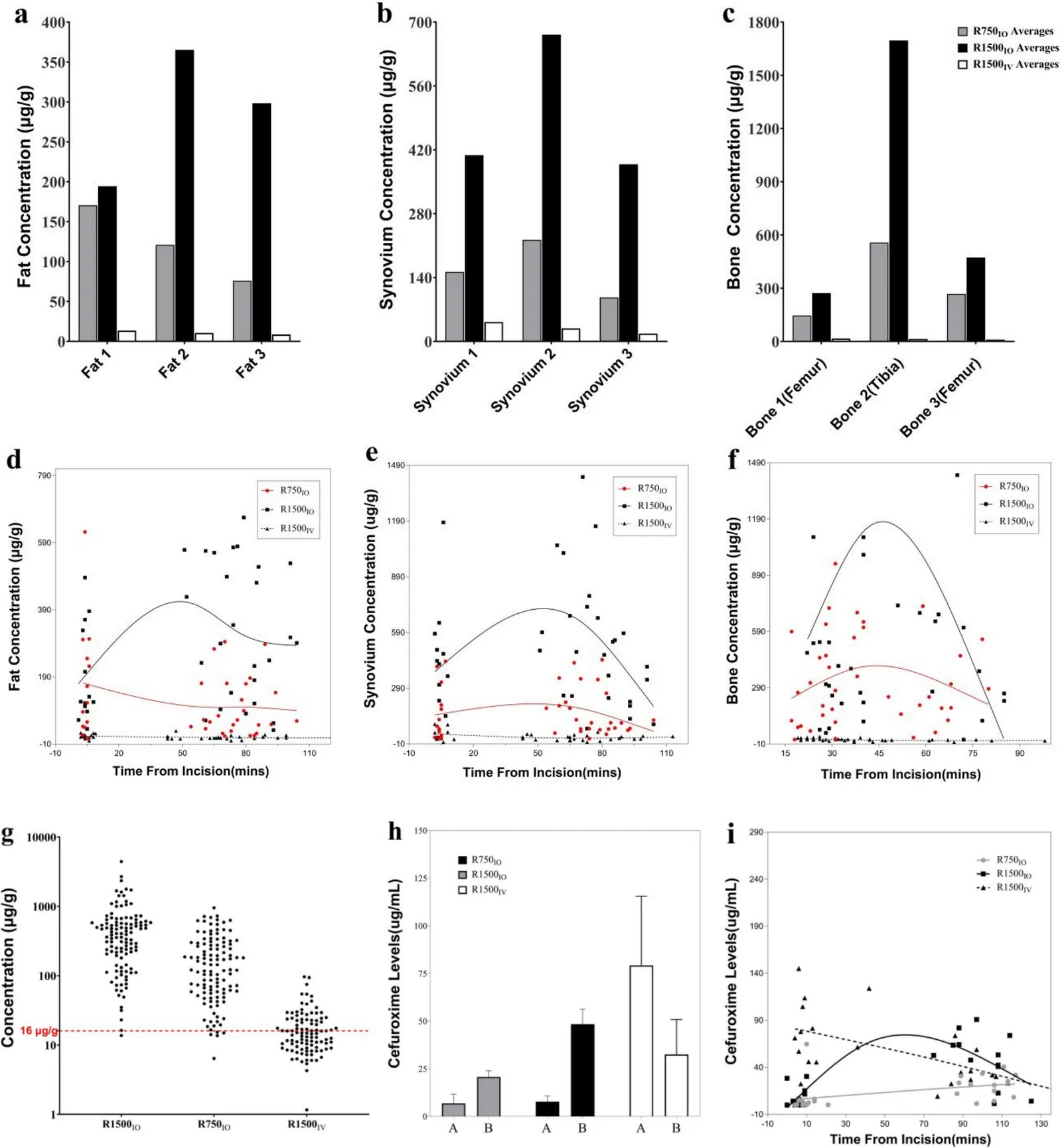
Comparison of mean cefuroxime concentrations in fat (**a**), synovium (**b**), and bone tissue (**c**) samples among the R750_IO_, R1500_IO_, and R1500_IV_ groups. Time–concentration profiles of cefuroxime in fat (**d**), synovium (**e**), and bone tissue (**f**) samples. Comparison of the overall distribution of cefuroxime concentration values in fat, synovium, and bone across the three patient groups; tissue cefuroxime concentrations are expressed as log10 values (**g**). Mean systemic cefuroxime concentrations in blood sample A, collected after skin incision, and blood sample B, collected after tourniquet release (**h**). Time–concentration profiles of systemic cefuroxime concentrations in blood samples across the three groups are shown in (**i**)

Cefuroxime concentrations in all fat, synovium, and bone samples were significantly higher in the R750_IO_ and R1500_IO_ groups than in the R1500_IV_ group (all *p* < 0.05) (Table 3 and Fig. 3a–f). According to the European Committee on Antimicrobial Susceptibility Testing (EUCAST) database, the cefuroxime epidemiological cutoff value (ECOFF), which is derived from the population minimum inhibitory concentration (MIC) distribution, is 4 μg/mL for Staphylococcus aureus [39]. Based on cefuroxime pharmacodynamic considerations, available antimicrobial susceptibility data, and the rationale described in detail in our previous study [22], a value of 16 μg/g was prespecified as a pharmacologically and microbiologically informed exploratory tissue-concentration benchmark, numerically corresponding to four times this referenced ECOFF. Because the EUCAST reference value is expressed as a fluid concentration in μg/mL, whereas tissue concentrations were measured in μg/g, the 16 μg/g value should be regarded as an operational benchmark rather than as a strict pharmacokinetic equivalence. In the R1500_IV_ group, 59.3% (64/108) of tissue samples were below the prespecified exploratory 16 μg/g benchmark. In contrast, 95.7% (112/117) and 99.1% (116/117) of tissue samples in the R750_IO_ and R1500_IO_ groups, respectively, achieved this level (Fig. 3g).

**Table 3:** Comparison of three groups in terms of sample concentrations of cefuroxime at each sample point

**Sample**	**R750**_**IO**_** (*n* = 13)**		**R1500**_**IO**_** (*n* = 13)**		**R1500**_**IV**_** (*n* = 12)**		***p*-value**^b^	***p*-value**^c^	***p*-value**^d^
	**Time, min (SD)**^a^	**Concentration (SD)**	**Time, min (SD)**^a^	**Concentration (SD)**	**Time, min (SD)**^a^	**Concentration (SD)**			
**Fat sample**									
1	4.4 (1.1)	170.4 (171.9)	4.3 (1.8)	194.3 (145.4)	5.3 (2.4)	13.5 (6.9)	0.39	< 0.001^e^	< 0.001^e^
2	67.0 (8.0)	120.9 (95.7)	68.7 (11.5)	365.3 (200.7)	63.6 (18.8)	10.4 (7.4)	< 0.001^e^	< 0.001^e^	0.001^e^
3	84.3 (8.9)	75.9 (74.7)	86.5 (12.1)	298.4 (206.7)	82.1 (22.1)	8.7 (4.2)	0.001^e^	< 0.001^e^	< 0.001^e^
**Synovium sample**									
1	4.3 (1.3)	152.3 (147.6)	4.5 (1.8)	407.9 (305.3)	4.9 (2.8)	42.6 (31.5)	0.016^e^	< 0.001^e^	0.035^e^
2	66.7 (8.1)	222.4 (134.1)	68.8 (11.4)	672.0 (398.2)	63.7 (18.8)	28.6 (14.8)	0.002^e^	< 0.001^e^	< 0.001^e^
3	84.1 (8.8)	96.1 (77.2)	86.8 (12.5)	388.2 (214.7)	79.6 (16.4)	17.0 (9.4)	< 0.001^e^	< 0.001^e^	< 0.001^e^
**Bone sample**									
1	26.1 (8.4)	146.0 (108.4)	30.8 (5.1)	272.0 (153.9)	26.8 (7.3)	16.2 (7.7)	0.024^e^	< 0.001^e^	0.001^e^
2	31.2 (7.1)	557.2 (182.5)	34.3 (7.3)	1695.9 (1035.3)	29.9 (5.8)	13.6 (7.4)	< 0.001^e^	< 0.001^e^	< 0.001^e^
3	65.1 (8.2)	267.2 (202.2)	67.5 (13.0)	472.8 (380.7)	61.7 (17.5)	10.0 (4.1)	0.10	< 0.001^e^	< 0.001^e^
**Blood sample**^**f**^									
A	9.7 (4.7)	6.8 (17.5)	5.5 (3.2)	7.7 (10.7)	13.7 (12.3)	79.2 (36.3)	0.92	< 0.001^e^	< 0.001^e^
B	24.5 (5.4)	20.7 (11.9)	21.8 (6.6)	48.4 (28.3)	24.7 (7.2)	32.5 (18.4)	0.005^e^	0.11	0.067

*SD* standard deviation

^a^The time of sample collection is calculated in minutes after skin incision for all except blood sample B

^b^Comparison of concentration differences between the R750_IO_ group and the R1500_IO_ group

^c^Comparison of concentration differences between the R1500_IO_ group and the R1500_IV_ group

^d^Comparison of concentration differences between the R750_IO_ group and the R1500_IV_ group

^e^The differences between groups are statistically significant

^f^A, Systemic blood samples collected after skin incision; B, blood samples collected after tourniquet release, with collection time calculated in minutes after deflation. Tissue cefuroxime concentrations are expressed in μg/g, whereas blood cefuroxime concentrations are expressed in μg/mL

The cefuroxime concentration in blood sample A collected at the start of surgery was significantly lower in the R1500_IO_ and R750_IO_ groups than in the R1500_IV_ group (*p* < 0.05) (Table 3, Fig. 3h). However, the cefuroxime concentration in blood sample B collected after tourniquet release was higher in the R1500_IO_ group than in the R750_IO_ and R1500_IV_ groups, with the difference between the R1500_IO_ and R750_IO_ groups being statistically significant (*p* = 0.005) (Table 3, Fig. 3h).

### Intraoperative changes in patient vital signs

Both the R750_IO_ and R1500_IO_ groups successfully completed the intraosseous administration of cefuroxime. All patients in these groups maintained stable vital signs during the intervention and 5–10 min after tourniquet release. No statistically significant differences (*p* > 0.05) were observed in the changes in vital signs among the three groups at the following time points: 5–10 min after intraosseous administration in the R750_IO_ and R1500_IO_ groups; 5–10 min after skin incision in the R1500_IV_ group; and 5–10 min after tourniquet release in all three groups (Table 4).

**Table 4:** Comparison of three groups in terms of changes in the patient's vital signs

Variable	R750_IO_ (*n* = 13)	R1500_IO_ (*n* = 13)	R1500_IV_ (*n* = 12)	*p*-value
5–10 min after IORA or incision*				
Temperature, ℃ (SD)	− 0.2 (0.5)	− 0.1 (0.5)	− 0.1 (0.5)	0.85
Respiration, times/minutes (SD)	− 4.8 (1.6)	− 5.5 (1.1)	− 4.9 (1.7)	0.32
Heart rate, times/minutes (SD)	− 19.7 (8.1)	− 18.2 (12.1)	− 20.3 (6.5)	0.87
Systolic blood pressure, mmHg (SD)	− 13.9 (20.6)	− 9.9 (29.1)	− 23.9 (24.5)	0.37
Diastolic blood pressure, mmHg (SD)	− 10.9 (10.8)	− 11.0 (15.9)	− 22.0 (13.6)	0.08
5–10 min after tourniquet release*				
Temperature, ℃ (SD)	− 0.4 (0.4)	− 0.2 (0.3)	− 0.2 (0.4)	0.20
Respiration, times/minutes (SD)	− 5.5 (1.5)	− 5.2 (0.8)	− 5.1 (1.5)	0.50
Heart rate, times/minutes (SD)	− 18.9 (9.6)	− 15.8 (12.7)	− 18.4 (8.8)	0.73
Systolic blood pressure, mmHg (SD)	− 26.6 (21.5)	− 24.5 (21.6)	− 28.3 (24.0)	0.91
Diastolic blood pressure, mmHg (SD)	− 20.4 (11.5)	− 23.1 (10.0)	− 24.2 (10.5)	0.83

*SD* standard deviation

^*^The difference between this time point and the vital signs measured at rest upon hospital admission

### Laboratory findings and complications

No statistically significant differences were observed among the R750_IO_, R1500_IO_, and R1500_IV_ groups in the changes of ESR, CRP, and IL-6 (postoperative days 1 and 3) or in other laboratory values (*p* > 0.05) (Table 5). No complications occurred within three months postoperatively in any of the groups, including those related to the intraosseous administration, wound or infection-related complications, or adverse drug reactions associated with cefuroxime.

**Table 5:** Comparison of three groups in terms of changes in laboratory findings

**Variable**	**R750**_**IO**_ **(*n* = 13)**	**R1500**_**IO**_ **(*n* = 13)**	**R1500**_**IV**_ **(*n* = 12)**	*p*-value*
**Postoperative day 1**				
CRP, mg/L (SD)	10.2 (10.4)	14.3 (13.5)	10.4 (12.4)	0.47
ESR, mm/h (SD)	3.8 (7.6)	2.8 (4.3)	5.0 (13.3)	0.80
IL-6, pg/mL (SD)	29.5 (21.2)	25.8 (18.2)	27.3 (18.4)	0.93
WBC, × 10^9^/L (SD)	6.0 (3.1)	5.7 (1.1)	4.9 (1.8)	0.40
Hb, g/L (SD)	− 2.9 (7.0)	− 5.7 (4.9)	− 8.5 (7.9)	0.13
PLT, × 10^9^/L (SD)	4.2 (32.7)	− 4.5 (22.1)	− 8.6 (34.3)	0.56
N, % (SD)	35.1 (10.5)	34.3 (7.1)	36.2 (13.3)	0.90
ALT, U/L (SD)	− 0.3 (6.4)	− 1.1 (7.8)	− 0.2 (3.4)	0.87
AST, U/L (SD)	− 0.1 (7.1)	1.0 (5.5)	1.6 (3.9)	0.76
DB, μmol/L (SD)	0.7 (1.5)	3.8 (7.3)	0.7 (1.0)	0.37
TB, μmol/L (SD)	1.3 (4.0)	3.1 (8.0)	1.1 (4.2)	0.62
Alb, g/L (SD)	− 1.3 (2.8)	− 1.8 (2.0)	− 2.5 (1.9)	0.42
Cr, μmol/L (SD)	0.6 (10.4)	− 0.7 (5.0)	− 2.3 (5.8)	0.64
Ur, mmol/L (SD)	− 0.3 (1.5)	− 0.2 (1.2)	− 0.5 (1.6)	0.89
PT, seconds (SD)	0.1 (1.8)	1.8 (2.0)	0.4 (1.6)	0.056
APTT, seconds (SD)	− 1.8 (2.3)	− 2.5 (5.4)	− 1.2 (3.8)	0.73
FIB, g/L (SD)	0.3 (0.5)	0.3 (0.6)	0.2 (0.5)	0.72
D-D, µg/L (SD)	1285.0 (951.2)	2789.5 (2467.9)	979.8 (703.1)	0.07
**Postoperative day 3**				
CRP, mg/L (SD)	44.7 (34.9)	35.7 (30.2)	40.4 (33.1)	0.74
ESR, mm/h (SD)	21.8 (15.7)	12.8 (15.4)	14.8 (10.0)	0.24
IL-6, pg/mL (SD)	22.5 (18.5)	14.0 (8.2)	21.4 (16.8)	0.44
WBC, × 10^9^/L (SD)	1.8 (2.7)	3.1 (1.9)	1.7 (1.5)	0.20
Hb, g/L (SD)	− 24.0 (27.7)	− 25.2 (6.6)	− 25.0 (6.3)	0.98
PLT, × 10^9^/L (SD)	− 36.5 (58.6)	− 29.1 (24.1)	− 38.3 (23.1)	0.50
N, % (SD)	10.0 (19.9)	16.8 (8.5)	15.5 (10.7)	0.76
ALT, U/L (SD)	− 3.0 (8.6)	− 1.7(9.7)	6.7 (22.1)	0.49
AST, U/L (SD)	− 2.6 (6.6)	− 0.0 (3.5)	4.3 (15.0)	0.45
DB, μmol/L (SD)	0.1 (1.0)	− 0.2 (1.4)	1.0 (1.4)	0.092
TB, μmol/L (SD)	− 0.5 (3.9)	− 1.7 (3.8)	0.5 (3.2)	0.33
Alb, g/L (SD)	− 7.3 (10.0)	− 5.2 (2.3)	− 5.3 (1.9)	0.72
Cr, μmol/L (SD)	− 13.1 (42.5)	0.3 (6.1)	− 2.7 (5.5)	0.36
Ur, mmol/L (SD)	− 1.5 (4.1)	0.7 (2.3)	− 0.6 (1.7)	0.27
PT, seconds (SD)	− 2.6 (4.1)	− 0.2 (2.2)	− 1.1 (1.2)	0.25
APTT, seconds (SD)	− 2.8 (8.6)	− 1.6 (5.2)	− 1.1 (4.2)	0.98
FIB, g/L (SD)	1.4 (1.8)	1.6 (0.9)	1.8 (1.3)	0.82
D-D, µg/L (SD)	577.6 (351.2)	1048.2 (1064.1)	682.7 (886.2)	0.58

*CRP* C-reactive protein, *ESR* erythrocyte sedimentation rate, *IL-6* interleukin-6, *WBC* white blood cell count, *Hb* hemoglobin, *PLT* platelet count, *N* neutrophil percentage, *ALT* alanine aminotransferase, *AST* aspartate aminotransferase, *DB* direct bilirubin, *TB* total bilirubin, *Alb* albumin, *Cr* creatinine, *Ur* urea, *PT* prothrombin time, *APTT* activated partial thromboplastin time, *FIB* fibrinogen, *D-D* D-dimer, *SD* standard deviation

^*^Comparison of differences in changes in indicators among the R750_IO_ group, R1500_IO_ group, and R1500_IV_ group;

Apart from antibiotic administration time, no significant overall differences were observed among the three groups in baseline demographic characteristics, intraoperative vital-sign changes, or postoperative laboratory and inflammatory-marker outcomes; therefore, no post hoc pairwise comparisons or Bonferroni adjustments were performed for these variables.

## Discussion

This randomized trial demonstrated that despite the use of multiple pins penetrating the intramedullary canal and longer operative and tourniquet times, preoperative stand-alone intraosseous cefuroxime produced substantially higher periarticular tissue concentrations than intravenous administration across the prespecified operative sampling interval in RA-TKA. Both intraosseous regimens were successfully incorporated into the robotic workflow, while the 750 mg regimen achieved high local tissue concentrations with lower systemic concentrations than the 1,500 mg intraosseous regimen. These findings support the technical feasibility of intraosseous cefuroxime administration in RA-TKA, demonstrate dose-related differences in its concentration profile, and identify 750 mg as a reasonable candidate dose for further clinical evaluation.

Previous studies have shown that the efficacy of perioperative prophylactic antibiotics should be assessed using both plasma and local tissue pharmacokinetic profiles [40]. Based on cefuroxime pharmacodynamic considerations, available antimicrobial susceptibility data, and the rationale described in detail in our previous study [22], a value of 16 μg/g—an operational benchmark numerically corresponding to four times the referenced cefuroxime ECOFF of 4 μg/mL for Staphylococcus aureus—was prespecified as an exploratory tissue-concentration benchmark [39]. In the present study, nine local tissue specimens were collected from each patient across multiple prespecified operative stages, providing a detailed stage-specific assessment of local cefuroxime exposure using the same sampling framework as our previous conventional-TKA trial, in which intraosseous cefuroxime produced higher measured tissue concentrations than intravenous administration [22]. The potential clinical relevance of this pharmacokinetic profile is indirectly supported by clinical experience with intraosseous vancomycin in primary TKA, which has been shown to achieve higher local tissue vancomycin concentrations while reducing systemic vancomycin concentrations [14–16]. An initial retrospective study of 1,060 primary TKAs reported a lower 90-day PJI rate with intraosseous rather than intravenous vancomycin, and a subsequent updated analysis of the expanded same institutional cohort of 1,923 TKAs demonstrated lower PJI rates at 90 days, one year, and two years [1, 4]. Their findings therefore support the biological plausibility that increased local antibiotic exposure with reduced systemic exposure may have clinical relevance. However, these findings should be extrapolated cautiously, as they evaluated intraosseous vancomycin in combination with intravenous cefazolin and were not conducted in RA-TKA. Accordingly, the present study provides an initial pharmacokinetic evaluation of preoperative stand-alone intraosseous cefuroxime within the distinct workflow of RA-TKA.

A robotic-specific concern was whether the proximity of the intraosseous access site to the tibial tracker-pin sites might interfere with regional drug delivery. The medullary cavity is a highly vascular structure, with a pressure of approximately 20–30 mmHg, and functions as a non-collapsible microvenous network [32, 33]. Intraosseous infusion uses this network to facilitate rapid, pressure-dependent regional drug delivery [32, 33]. Its efficiency may be influenced by the anatomical and vascular characteristics of the bone as well as by changes in resistance between the medullary cavity and the vascular system [34, 35]. Because both the IORA access site and the tibial tracker-pin sites were located in the proximal tibial region, insertion and subsequent removal of the pins could theoretically affect fluid egress, medullary pressure, flow resistance, or regional drug distribution [31]. In the present workflow, cefuroxime was administered before tibial tracker-pin insertion, and the pins remained seated within the transcortical tracts until implant placement; therefore, no open pin tract was present during administration. We found that IORA was completed in all patients, and no visible leakage from either the intraosseous access site or the tracker-pin sites was observed intraoperatively, including after tracker-pin insertion and removal. Cefuroxime concentrations in the two earliest tissue specimens collected within minutes of IORA exceeded the exploratory 16 μg/g tissue-concentration benchmark, indicating rapid regional distribution within the operative limb. This finding was consistent with previous studies of IORA cefazolin or vancomycin in patients undergoing TKA [11, 14, 15, 41]. Taken together, the successful administration, absence of visible leakage, and high tissue concentrations observed across the prespecified sampling points support the technical feasibility of incorporating IORA into the robotic workflow and suggest that tracker-pin placement did not obviously compromise regional cefuroxime delivery. However, because the study did not directly compare conditions with and without tracker pins, an independent pharmacokinetic effect of tracker-pin insertion and removal cannot be excluded [31, 32].

The duration of regional antibiotic exposure represents another important consideration in RA-TKA. Compared with conventional TKA, RA-TKA generally requires longer operative times, which may exceed one hour and reach 2–3 h during the learning curve [42, 43]. Tourniquet inflation also restricts the entry of intravenously administered antibiotics into the operative limb and has been shown to reduce periarticular tissue antibiotic concentrations [13]. The longer operative and tourniquet times associated with RA-TKA therefore raised uncertainty as to whether the high local concentrations achieved with intraosseous cefuroxime could be maintained throughout the robotic procedure [36, 44]. In the present study, both the 750 mg and 1,500 mg IORA regimens produced high measured cefuroxime concentrations at all prespecified tissue-sampling points, including those obtained during the later operative stages and after tourniquet release. In contrast, intravenous infusion of 1,500 mg cefuroxime produced substantially lower tissue concentrations, with many samples below the exploratory 16 μg/g benchmark. Cefuroxime concentrations in fat, synovium, and bone in the R750_IO_ group were 9–13, 4–8, and 9–41 times higher, respectively, than those in the R1500_IV_ group, while the R1500_IO_ group produced even higher concentrations. These findings indicate that high measured regional cefuroxime concentrations were present throughout the operative interval examined in this study.

We observed that tissue cefuroxime concentrations after IORA were higher in RA-TKA than in our previously reported conventional-TKA cohort [22]. At both the 750 mg and 1,500 mg doses, mean tissue concentrations were higher in RA-TKA at seven of the nine matched sampling points, particularly in fat and synovial samples collected later in the procedure. This difference was especially pronounced in the first distal femoral cancellous bone specimen. After 750 mg IORA, the mean concentration was 146.0 μg/g in RA-TKA compared with 25.3 μg/g in the conventional-TKA cohort; after 1,500 mg IORA, the corresponding concentrations were 272.0 and 77.7 μg/g [22]. Preservation of the femoral medullary canal may partly explain this concentration pattern. In conventional TKA, the femoral intramedullary alignment rod is inserted before distal femoral osteotomy and specimen collection, whereas the central femoral canal remains intact during robotic bone preparation. Opening and instrumenting the canal may disturb medullary circulation, decompress the medullary compartment, and increase medullary bleeding, thereby increasing local fluid volume, tissue dilution, and drug washout [31, 45]. However, the first distal femoral bone specimen was also collected later in RA-TKA than in conventional TKA, allowing a longer interval for cefuroxime distribution. The observed differences may therefore reflect a combination of preserved medullary integrity, reduced canal-associated bleeding and dilution, and longer drug-distribution time. Nevertheless, the two cohorts were recruited during different periods and were not randomized directly against each other. These cross-cohort findings should therefore be interpreted cautiously. Although they used the same doses, anatomical sampling sites, operative sampling framework, specimen-processing procedures, and LC–MS/MS method, the comparison should be regarded as descriptive rather than as evidence of a causal effect of the robotic workflow.

With respect to systemic cefuroxime concentrations, we also observed route- and dose-related differences. The initial blood concentrations were significantly lower in both intraosseous groups than in the R1500_IV_ group. After tourniquet release, the R1500_IO_ group had a higher mean blood concentration than the R750_IO_ and R1500_IV_ groups, although the difference was statistically significant only between the two intraosseous-dose groups. These systemic concentration findings should be interpreted in relation to differences in administration route, administration duration, tourniquet isolation and release, and sampling timing [13]. Intravenous cefuroxime was administered over a substantially longer period than the intraosseous regimens, and the intervals between antibiotic administration and collection of blood sample A were not identical among the groups. The higher initial systemic concentration in the R1500_IV_ group therefore likely reflects direct systemic administration and longer time for systemic distribution, whereas intraosseously administered cefuroxime was initially retained within the tourniquet-isolated operative limb [13, 36]. This pattern is consistent with previous IORA studies showing lower early systemic concentrations before tourniquet release [5]. The systemic cephalosporin concentrations decline over time in the intravenous group. This may be because, as the operation progresses, the drug is distributed systemically and undergoes further metabolism. Notably, the R1500_IO_ regimen produced higher tissue concentrations than the R750_IO_ regimen but was also associated with a significantly higher post-tourniquet-release blood concentration. Previous randomized evidence has similarly shown that higher-dose intraosseous cephalosporin administration can produce tissue concentrations several-fold higher than intravenous administration, but these levels may be excessively high [46]. The combination of high local concentrations and lower systemic concentrations therefore supports 750 mg intraosseous cefuroxime as a reasonable candidate dose for further evaluation in RA-TKA, although the present study does not establish it as the preferred or clinically optimal prophylactic dose.

In this study, “stand-alone” refers only to the randomized preoperative prophylactic regimen, with no additional systemic antibiotic administered before incision or intraoperatively [18]. Our results showed no significant between-group differences in early postoperative inflammatory markers after preoperative intraosseous or intravenous administration of cefuroxime. Furthermore, no infections were observed within three months in any group, and no significant intergroup differences were observed in renal and hepatic function or coagulation parameters. Although these secondary clinical outcomes were exploratory and underpowered —and were assessed in the context of a shared postoperative intravenous antibiotic regimen—they were not used to support conclusions regarding safety or infection-prevention efficacy. Taken together, these findings support the technical feasibility of using intraosseous cefuroxime as the sole antibiotic administered before incision and intraoperatively in RA-TKA. The study confirms the reproducibility and technical feasibility of a previously studied cefuroxime regimen within the robotic workflow and, unexpectedly, demonstrates higher tissue cefuroxime concentrations at most matched sampling points than those in our previously reported conventional TKA cohort. However, because total cefuroxime concentrations were measured in tissue homogenates at discrete time points rather than through continuous monitoring of unbound interstitial drug, the proportion of samples at or above this benchmark should be interpreted as a descriptive pharmacokinetic outcome rather than as direct evidence of continuous free-drug pharmacodynamic target attainment. Furthermore, as the study was not powered to evaluate PJI or wound outcomes, whether the observed pharmacokinetic advantages translate into improved clinical prophylaxis remains to be determined.

The main potential limitation of this study is its small sample size. Although determined by an a priori power analysis for cefuroxime tissue concentration, this sample size was underpowered to detect statistically significant differences in secondary clinical outcomes, including PJI, wound complications, and inflammatory marker levels. Furthermore, this study did not enroll patients at high risk of infection. Larger, adequately powered studies are required to confirm these pharmacokinetic findings and determine whether the 750 mg intraosseous regimen provides clinically meaningful prophylactic benefit.

## Conclusion

Despite the use of multiple intramedullary pins and longer operative time in robot-assisted TKA, a single intraosseous administration of cefuroxime still achieved high local tissue concentrations. Compared with conventional intravenous infusion of 1,500 mg, intraosseous administration—whether 750 mg or 1,500 mg—resulted in higher local tissue concentrations and significantly lower initial systemic levels. Notably, while the 1,500 mg intraosseous dose was associated with higher tissue concentrations that may exceed optimal levels, the 750 mg dose offered lower systemic concentrations. These findings support the technical feasibility of intraosseous cefuroxime in RA-TKA and identify 750 mg as a reasonable candidate dose for further clinical evaluation; adequately powered studies are required to determine whether this concentration profile translates into improved infection-prevention outcomes.

## Data Availability

The datasets generated and/or analyzed during the current study are available from the corresponding author on reasonable request.
